# Assessment of Pancreatic Lipase Activity Using a Quantitative and a Qualitative Assay in Dogs with Chronic Kidney Disease

**DOI:** 10.3390/ani16091282

**Published:** 2026-04-22

**Authors:** Dimitra Pardali, Rafailia Karaiosif, Argyrios Ginoudis, Katerina K. Adamama-Moraitou, Zoe Polizopoulou

**Affiliations:** 1 Diagnostic Laboratory, Department of Clinical Studies, School of Veterinary Medicine, Faculty of Health Sciences, Aristotle University of Thessaloniki, Stavrou Voutira 11str, 54636 Thessaloniki, Greece; agkinou@vet.auth.gr (A.G.); poliz@vet.auth.gr (Z.P.); 2 Companion Animal Clinic (Unit of Medicine), Department of Clinical Studies, School of Veterinary Medicine, Faculty of Health Sciences, Aristotle University of Thessaloniki, Stavrou Voutira 11str, 54636 Thessaloniki, Greece; rafailiak@yahoo.com (R.K.); kadamama@vet.auth.gr (K.K.A.-M.)

**Keywords:** chronic kidney disease, dog, pancreatic lipase, DGGR assay, SNAP cPL, acute pancreatitis

## Abstract

Chronic kidney disease (CKD) in dogs is associated with increased serum pancreatic lipase activity, complicating the diagnosis of acute pancreatitis (AP). This prospective study evaluated pancreatic lipase activity measured using a quantitative DGGR assay in dogs with CKD while excluding cases with clinical or imaging evidence of AP, and the DGGR results were compared with the qualitative SNAP cPL test. Twenty-five dogs with IRIS stage 1–4 CKD were enrolled. The DGGR results were within the reference range in 52% of dogs and elevated in 48%, whereas the SNAP cPL results were abnormal in 72% of cases. Discordance between tests was common, with several dogs showing abnormal SNAP cPL results despite normal DGGR values, a difference that was statistically significant (*p* = 0.016). The DGGR results revealed significant differences between normal and abnormal values relative to serum creatinine and blood urea nitrogen concentration, as well as total serum lipase activity, suggesting an influence of impaired renal function on pancreatic lipase. No significant association was observed between CKD stage and either DGGR or SNAP cPL results. These findings indicate that pancreatic lipase activity may be increased in dogs with CKD independently of AP and that SNAP cPL may overestimate pancreatic enzyme elevation in this population. DGGR appears to be more reliable for excluding AP in dogs with CKD, although positive results should be interpreted cautiously in conjunction with clinical and imaging findings.

## Simple Summary

Chronic kidney disease (CKD) is common in older dogs and can affect the interpretation of blood tests used to diagnose other diseases, including acute pancreatitis. Pancreatic lipase is an enzyme commonly measured to help identify pancreatitis, but its levels may increase in dogs with CKD, even when pancreatic disease is not present. This study evaluated pancreatic lipase activity in dogs with CKD using a quantitative test (DGGR assay) and compared the results with a commonly used rapid screening test (SNAP cPL). Twenty-five dogs with CKD and no evidence of pancreatitis were included. The two tests often disagreed, with SNAP cPL frequently indicating abnormal results when DGGR values were normal. Pancreatic lipase activity measured using DGGR was also associated with the severity of kidney dysfunction. These findings suggest that CKD alone can cause increased pancreatic lipase activity, and that SNAP cPL may overestimate pancreatic involvement in dogs with CKD. The DGGR assay may be more useful for ruling out pancreatitis in dogs with CKD, although test results should always be interpreted alongside clinical and imaging findings.

## 1. Introduction

Chronic kidney disease (CKD) is a progressive and irreversible condition characterized by a gradual loss of renal function, primarily affecting older dogs, resulting from a complex interplay of genetic, environmental, and infectious factors [1]. CKD is defined by the presence of structural or functional kidney abnormalities persisting for more than 3 months and may be clinically manifested by polyuria, polydipsia, lethargy, appetite loss, and weight loss [2,3]. Epidemiological studies report a prevalence ranging from approximately 0.5% to 25%, particularly among geriatric dogs, making CKD one of the most common renal disorders in canine patients [4,5].

CKD has been increasingly associated with gastrointestinal disorders, including pancreatitis. Acute pancreatitis (AP) is a common condition in dogs and is characterized by clinical signs such as vomiting, abdominal pain, and lethargy. Dogs with CKD may be predisposed to pancreatic enzyme alterations due to impaired renal function, leading to diagnostic challenges when interpreting serum pancreatic enzyme activities [6,7]. Elevated serum lipase activity, in particular, has been documented in dogs with CKD in the absence of primary pancreatic disease, limiting its diagnostic specificity [8]. Consequently, differentiation between true pancreatic pathology and secondary alterations in pancreatic lipase activity due to CKD is of clinical importance.

Pancreatic lipase is a crucial enzyme in lipid digestion, and its measurement is widely used for the diagnosis of AP in dogs [9] since it is considered a highly sensitive biomarker, with high concordance when compared to pancreatic biopsy results and other immunoenzymatic methods [10]. The SNAP cPL (Canine Pancreatic Lipase Kit, IDEXX Laboratories Inc., Westbrook, ME, USA) is a commonly used point-of-care qualitative test designed primarily as an exclusion tool and not as a confirmation test for AP [11]. An alternative test is the 1,2-o-dilauryl-rac-glycero-3-glutaric acid-(6′-methylresorufin) ester (DGGR) assay, which provides a quantitative measurement of pancreatic lipase activity and has been suggested to offer improved diagnostic resolution in certain clinical contexts [12,13,14]. However, data regarding DGGR performance in dogs with CKD and its agreement with SNAP cPL in this population remain limited.

Systemic diseases, including renal and hepatic disorders, hyperadrenocorticism, and portal hypertension, have been associated with alterations in serum lipase activity, raising concerns about the accuracy of pancreatic lipase assays in dogs with CKD [14,15,16,17,18,19].

The primary objective of this prospective study was to investigate the variation of pancreatic lipase activity using the DGGR assay in dogs diagnosed with CKD, excluding cases of AP. Additionally, the aim of this study was to compare the results of the DGGR assay with those obtained from the SNAP cPL test in the same group of animals. By assessing the degree of agreement between these two diagnostic methods, we sought to determine whether DGGR could serve as a reliable alternative to SNAP cPL for evaluating pancreatic lipase activity in dogs with CKD. Identifying potential discrepancies between the two tests could provide valuable insights into their respective clinical applications and limitations, ultimately aiding veterinarians in making more informed diagnostic and treatment decisions.

## 2. Materials and Methods

### 2.1. Study Design and Animal Selection

This prospective study involved dogs presented to the Companion Animal Clinic, School of Veterinary Medicine, Faculty of Health Sciences, Aristotle University of Thessaloniki, Greece, over 1-year period. All dogs enrolled in the study were required to meet predefined inclusion criteria. Specifically, the dogs had to be adults (>1 year of age), irrespective of sex, and diagnosed with CKD stages 1 to 4, according to the International Renal Interest Society (IRIS) staging system (www.iris-kidney.com) [20], while being adequately hydrated at the time of evaluation. Exclusion criteria were: (a) administration of pharmaceutical agents previously associated with the development of AP within 2 weeks prior to inclusion, such as corticosteroids, cholinesterase inhibitors, potassium bromide, phenobarbital, l-asparaginase, estrogen, salicylates, azathioprine, thiazide diuretics, and vinca alkaloids [21]; (b) any history suggestive of current or previous AP, including recurrent, self-limiting episodes of intermittent anorexia; (c) the presence of major clinical signs compatible with AP: abdominal pain; fever; icterus; cardiovascular shock; multiorgan failure, (d) imaging findings (ultrasonographic, radiographic, or computed tomography) consistent with pancreatitis or liver or intestinal disease, (e) positive urine culture obtained aseptically via cystocentesis. Dogs receiving medications aimed at maintaining stable CKD—including angiotensin-converting enzyme inhibitors (ACEIs), proton pump inhibitors, phosphorus binders, and ferrous sulfate supplements—were not excluded from the study protocol.

### 2.2. Physical Examination

Each dog underwent a comprehensive physical examination, during which care was taken to recognize any clinical findings compatible with AP. Hydration status was monitored to assess whether the animal was adequately hydrated to allow the collection of the required blood samples for the study. In case of dehydration, sampling was withheld until the animal was adequately hydrated. Then, a comprehensive physical examination was repeated, and if the animal met the inclusion criteria, sampling was carried out. Additional parameters, such as body temperature, heart rate, heart rhythm, respiratory rate, and pulse character, were recorded. Mucous membrane colour was noted, and capillary refill time was measured. Blood pressure was recorded using the Doppler ultrasonography method.

### 2.3. Clinicopathologic Testing

Venous blood samples were taken after the animal was adequately hydrated, and the samples underwent biochemical blood serum tests, including concentration of total protein (g/dL), albumin (g/dL), urea nitrogen (mg/dL), creatinine (mg/dL), phosphorus (mg/dL), total calcium (mg/dL), glucose (mg/dL), cholesterol (mg/dL), triglycerides (mg/dL), total bilirubin (mg/dL) and the activity of alkaline phosphatase (U/L), alanine aminotransferase (U/L), and lipase (U/L) using an automated liquid chemistry analyzer (Vitalab Flexor E Chemistry Analyzer, Vital Scientific N.V., Dieren, The Netherlands). Furthermore, the concentrations of electrolytes—potassium (mEq/dL) and sodium (mEq/dL)—were measured using an automated electrolyte analyzer (Roche 9180 Electrolyte Analyzer, Roche Diagnostics, Mannheim, Germany). The remaining serum aliquots were collected and stored at −80 °C until further analysis (pancreatic lipase measurements).

All animals underwent a complete blood count (CBC) using an automated hematological analyzer (ADVIA 120 Hematology System, Siemens Healthcare Diagnostics, Tarrytown, NY, USA). The CBC results recorded included hematocrit (%), hemoglobin concentration (g/µL), total white blood cell count (cells/µL), and total platelet count (cells/µL).

Urine samples were collected via cystocentesis prior to the initiation of intravenous fluid administration at the time of presentation. The specific gravity of the urine was measured using a clinical refractometer (Atago Co., Ltd., Fukaya-shi, Japan), and pH, blood elements, glucose, ketone bodies, bilirubin, and protein were detected using a chromatometric strip (Medi-Test Combi 10 Vet, Macherey-Nagel, Neumann-Neander, Düren, Germany). Following the centrifugation of 5 mL of urine the sediment was examined microscopically for crystals, cellular elements, and microorganisms. Additionally, an aerobic culture was performed. The urinary protein and creatinine concentrations were measured using an automated liquid chemistry analyzer (Vitalab Flexor E Chemistry Analyzer, Vital Scientific N.V., Dieren, The Netherlands). The urine protein-to-creatinine (UPC) ratio was then calculated to evaluate the degree of proteinuria.

### 2.4. Measurement of Pancreatic Lipase Activity Using Quantitative DGGR Assay

Pancreatic lipase activity in blood serum samples was measured using the Eurolyser Solo Analyzer (Eurolyser Diagnostica GmbH, Salzburg, Austria). According to the manufacturer, normal pancreatic lipase values for dogs range from 0 to 125 U/L, and the measurement range of the analyzer extends from 0 to 300 U/L. In this assay, the activity of pancreatic lipase was determined using the enzymatic 1,2-o-dilauryl-rac-glycero-3-glutaric acid-(6′-methylresorufin) ester (DGGR) assay. In this quantitative assay, lipase, in the presence of bile acids, hydrolyzes the synthetic substrate 1,2-o-dilauryl-rac-glycero-3-glutaric acid-(6′-methylresorufin) ester into glycerol and methylresorufin ester, which then splits into glutaric acid and methylresorufin. The reaction’s specificity for pancreatic lipase is ensured by the combined activity of glylipase and bile acids, which excludes the involvement of other esterases or lipolytic enzymes. The resulting measured uptake is directly proportional to the pancreatic lipase activity in the sample.

### 2.5. Abdominal Imaging

All dogs included in the study underwent abdominal radiography and ultrasonographic examination of the abdomen. Only findings consistent with chronic kidney disease were considered acceptable on radiographic or ultrasonographic evaluation.

### 2.6. Determination of Pancreatic Lipase Activity Using Qualitative SNAP cPL Assay

The pancreatic lipase concentration was also measured using the qualitative SNAP cPL (Canine Pancreatic Lipase Kit, IDEXX Laboratories Inc., Westbrook, ME, USA) immunoreaction assay, a rapid point-of-care qualitative method for detecting pancreatic lipase that works as a quick screening test that confirms or excludes AP. The test was performed according to the manufacturer’s instructions. The result is either normal or abnormal based on the intensity of a colored dot that appears in the test well. If no dot appears or the color is less intense than the control, the pancreatic lipase activity is considered normal. If the color intensity is equal to or more intense than the control, the pancreatic lipase activity is considered abnormal.

### 2.7. Statistical Analysis

Descriptive statistics were used to summarize the data, including absolute and relative frequencies (%), measures of central tendency (means and medians), and measures of dispersion (range, interquartile range and standard deviation). Group comparisons based on central tendency were conducted using the Mann-Whitney (M-W) test. The χ^2^ test was applied to compare frequency distributions and assess associations between categorical variables. For both the M-W and *χ*^2^ tests, *p*-values were obtained via Monte Carlo simulation with 10,000 random samples [22], ensuring robustness even when standard test assumptions (random sampling, independent observations, symmetric distributions, and absence of extreme values) were not met. A significance level of α = 0.05 (*p* ≤ 0.05) was applied to all statistical tests. Analyses were performed using IBM SPSS v24, with the Exact Tests module for Monte Carlo simulations.

## 3. Results

### 3.1. Epidemiological Data

A total of 86 dogs known or suspected to have CKD were initially evaluated for inclusion in the study. Of these, 61 were excluded due to abdominal pain upon clinical examination (26 dogs), ultrasonographic and/or radiographic findings suggestive or compatible with AP (19 dogs), death during hospitalization before adequate hydration could be achieved to allow sampling (eight dogs), and/or the premature discontinuation of hospitalization at the owner’s request, resulting in failure to achieve adequate hydration prior to sampling (12 dogs). Twenty-five dogs diagnosed with CKD met the inclusion criteria and were ultimately included in the study. The population comprised 16/25 (64%) purebred and 9/25 (36%) mixed-breed dogs. Males accounted for 16/25 (64%) dogs, with 2/16 (12.5%) of them having been neutered, while females accounted for 9/25 (36%), with 1/9 (11.1%) having been neutered. The dogs’ ages ranged from 1.5 to 18 years, (median 7.2/IQ = Q75-Q25 6.4 years), while their body weight varied between 1.7 and 34.5 kg (median 19.81/IQ = Q75-Q25 10.2 kg).

### 3.2. Clinical Findings and Laboratory Results

The clinical examination findings and symptoms for all study dogs suffering from CKD are presented in Table 1. Clinical examination findings were recorded at the time of sampling. Blood pressure was measured in all study dogs, with values ranging from 75 to 275 mmHg (median 158.2; IQ = Q75-Q25 134.5 mmHg).

The complete blood count, serum biochemistry profile, and DGGR results for the 25 dogs with CKD that were enrolled in the study are summarized in Table 2 and Table 3. The pancreatic lipase activity measured via DGGR assay remained within the reference range (0–125 U/L) in 13/25 (52%) dogs but exceeded the upper normal limit in 12/25 (48%). Serum lipase activity and blood urea nitrogen (BUN) concentration were elevated in all animals. Creatinine concentration was also above normal in 24/25 (96%) dogs, with only one dog displaying a creatinine concentration of 1.2 mg/dL.

Additionally, all dogs underwent a quantitative SNAP cPL test, with results being normal in 7/25 (28%) and abnormal in 18/25 (72%) cases.

Urinalysis revealed that all dogs exhibited decreased urine specific gravity (reference value > 1.030). Blood, glucose, ketone bodies, and bilirubin were not detected in any urine sample. The microscopical examination of urine sediment was unremarkable in all samples. The UPC ratio was elevated in 23/25 dogs (92%), while only 2/25 (8%) remained normal. Based on urine culture, none of the 25 study dogs tested positive for urinary tract infection.

Following the IRIS guidelines, the dogs were classified into four CKD stages. Two out of 25 (8%) dogs were classified as stage 1, 6/25 (24%) as stage 2, 8/25 (32%) as stage 3, and 9/25 (36%) as stage 4. Table 4 depicts the variations in kidney function related biochemistry parameters along with the DGGR results and urinalysis findings at each of the CKD stages.

The association between DGGR and SNAP cPL is presented in Table 5. The results demonstrated that from the 13/25 dogs with a normal DGGR value, only seven dogs also had a normal SNAP cPL result, while the rest showed an abnormal SNAP cPL result despite a normal DGGR value. This difference was statistically significant (*χ*^2^ test, *p* = 0.016). Moreover, none of the dogs with an abnormal DGGR result had a normal SNAP cPL test.

Table 6 presents pancreatic lipase activity (normal and abnormal results) from both assays across the four CKD stages. The results indicate that DGGR correctly identified both dogs in stage 1 CKD as negative for AP, while in stages 2, 3, and 4, the dogs were nearly evenly distributed between normal and abnormal, leading to no statistically significant association between DGGR results and CKD stages (*χ*^2^ test, *p* = 0.671). Similarly, SNAP cPL correctly detected both stage 1 cases as normal, whereas the majority of dogs in later stages had abnormal results. No statistically significant association was observed between SNAP cPL results and CKD stage (*χ*^2^ test, *p* = 0.082).

**Figure 1:**
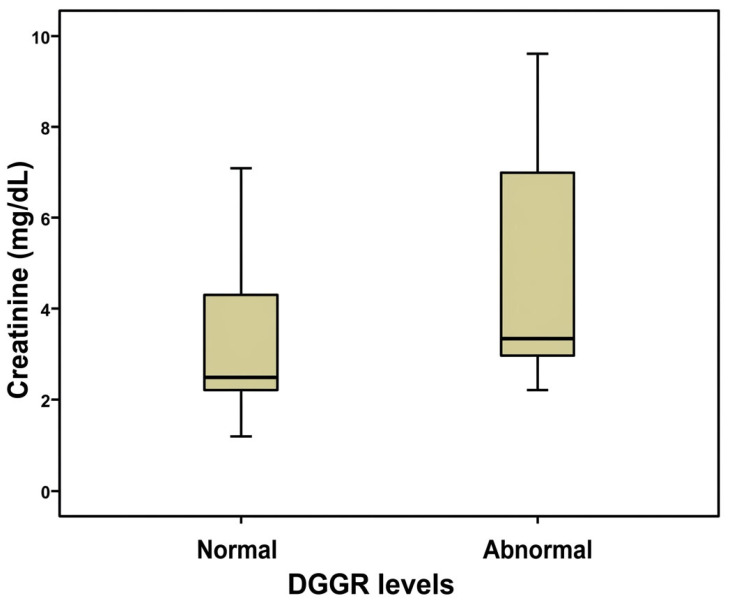
Comparison between normal and abnormal DGGR values measured via DGGR assay relative to serum creatinine concentration in 25 dogs with chronic kidney disease included in the study.

**Figure 2:**
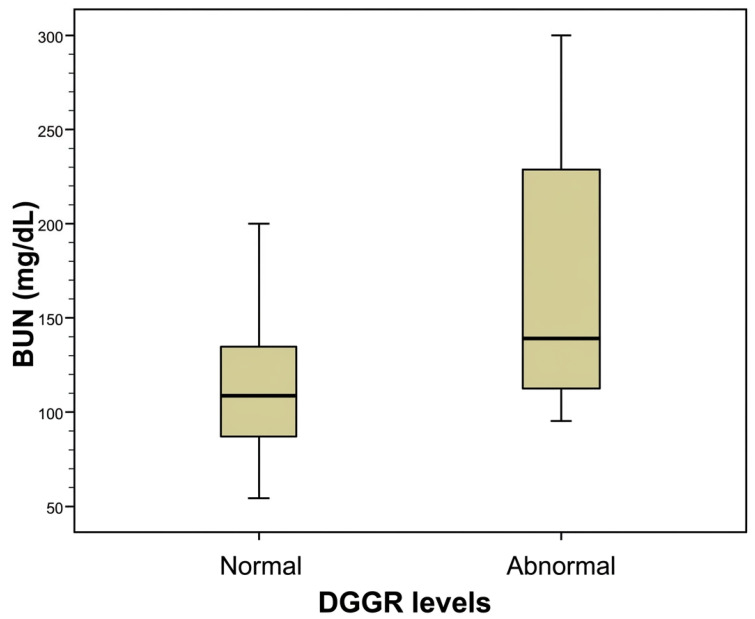
Comparison between normal and abnormal DGGR values measured via DGGR assay relative to blood urea nitrogen (BUN) concentration in 25 dogs with chronic kidney disease included in the study.

**Figure 3:**
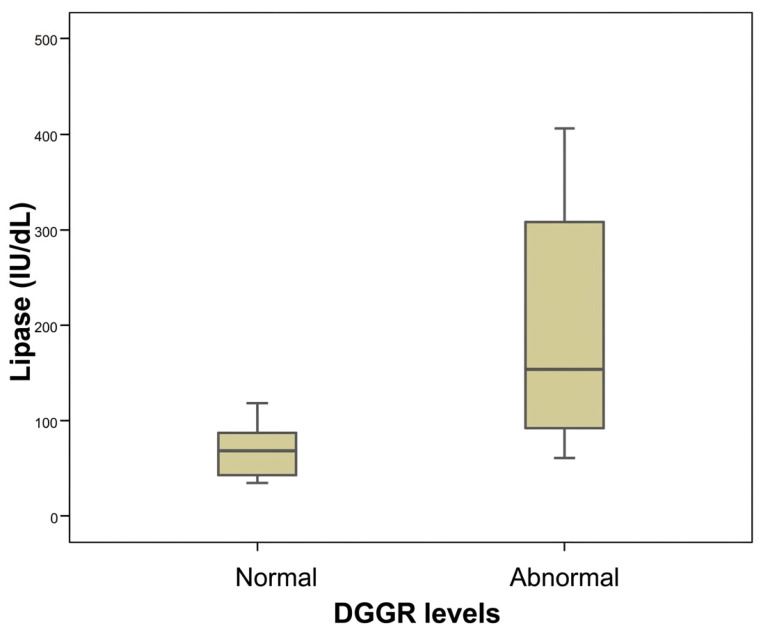
Comparison between normal and abnormal DGGR values measured via DGGR assay relative to serum lipase concentration in 25 dogs with chronic kidney disease included in the study.

Further investigation of the DGGR results revealed significant differences between normal and abnormal DGGR values relative to creatinine (Mann-Whitney test, *p* = 0.050, Figure 1) and BUN (Mann-Whitney test, *p* = 0.030, Figure 2) concertation, as well as relative to serum lipase activity (Mann-Whitney test, *p* < 0.001, Figure 3).

## 4. Discussion

The measurement of serum lipase activity has long been utilized in the diagnostic evaluation of AP in both veterinary and human medicine. Although increased lipase activity is commonly associated with pancreatic inflammation, several extra-pancreatic tissues and systemic disorders have been shown to contribute to elevated serum lipase activity, thereby limiting the specificity of this biomarker [8]. In dogs with naturally occurring or experimentally induced renal disease, increased serum lipase activity has been reported in the absence of clinical or histopathological evidence of AP [23,24].

In the present study, pancreatic lipase activity measured using the DGGR assay remained within the reference range (upper normal range 125 U/L) in approximately half of dogs with CKD, while the remaining animals exhibited increased values. This may be explained by the fact that although the DGGR assay exhibits improved specificity compared to older catalytic methods, the complete exclusion of interference from extra-pancreatic esterases and lipases is not possible [25]. This limitation may account for the increased DGGR values observed in some dogs without clinical or imaging evidence of AP, emphasizing the importance of assay-specific interpretation in dogs with concurrent systemic disease. The DGGR assay has been reported to demonstrate high sensitivity for AP, particularly when low cut-off values are applied, which is desirable when used as a screening or exclusion test [25]. One the other hand, raising the upper reference limit to 216 U/L has been shown to improve diagnostic accuracy [12,26].

A high proportion of dogs with CKD yielded abnormal SNAP cPL results. Notably, discordance between SNAP cPL and DGGR measurements was common, with several dogs exhibiting abnormal SNAP cPL results despite DGGR values within the reference range, which is a significant discrepancy. SNAP cPL has been reported to yield false positives, with up to 40% of dogs without AP compatible clinical and laboratory findings [27]. The SNAP cPL assay classifies all samples with pancreatic lipase concentrations ≥ 200 μg/L as abnormal, without distinguishing between equivocal and diagnostically significant values. In contrast, Spec cPL, a quantitative assay, incorporates a gray zone, with concentrations ≥ 400 μg/L considered consistent with AP [27]. This fundamental difference likely explains the higher apparent false-positive rate observed with SNAP cPL in dogs with CKD in the present study.

Although an official gray zone has not been established for the DGGR assay, individual biological variability comparable to that described for Spec cPL is likely [12]. Applying a gray zone (109–216 U/L) for DGGR improves agreement with Spec cPL [12]. If we re-evaluate our data using this threshold, only 7/25 dogs had values suggestive of AP. Using Cridge’s [26] gray zone (141–216 U/L), 7/25 were abnormal, 2/25 were equivocal, and 16/25 were normal. The high false-positive rate of SNAP cPL is due to its binary classification (≥200 µg/L as positive), whereas Spec cPL, which is a quantitative assay, recognizes a gray zone (200–399 µg/L) before classifying a dog as suffering from AP (≥400 µg/L [27]. Confirmation with Spec cPL is recommended for both DGGR [25] and SNAP cPL [27], as Spec cPL remains the most sensitive and specific test for AP [14,28]. Our findings suggest that DGGR is superior to SNAP cPL in excluding AP in dogs with CKD and in the future the application of interpretative thresholds may enhance the diagnostic reliability of DGGR particularly in dogs with CKD. Additionally, the use of DGGR instead of SNAP cPL facilitates better selection of cases for quantitative Spec cPL measurement, reducing hospitalization costs while enabling the faster and more targeted determination of the therapeutic protocol.

Total serum lipase activity was increased in all dogs included in this study, and a strong positive association was identified between total serum lipase activity and DGGR-measured pancreatic lipase. This finding suggests that elevations in total lipase activity in dogs with CKD parallel changes detected by the DGGR assay. This is partially consistent with earlier studies reporting increased serum lipase activity in dogs with CKD [23,24] or other conditions [29] but differ from those of another report where lipase activity remained within normal limits when measured using a catalytic method [8]. Such discrepancies are likely attributable to methodological differences among assays, as variations in substrates and reaction conditions influence their selectivity for pancreatic versus extra-pancreatic lipases [13].

The impact of azotemia on catalytic methods for determining serum lipase activity in blood serum, particularly due to a decrease in glomerular filtration rate, remains a topic of debate. In human CKD patients, lipase activity has been found to be elevated despite the absence of AP [30,31,32]. In CKD, dogs’ serum lipase levels were found elevated, although no correlation was observed with serum creatinine or urea nitrogen [24]. In dogs with suspected AP and CKD, mean lipase, amylase, and Spec cPL levels were significantly increased [33]. However, in experimentally induced CKD, lipase values did not correspond to the severity of azotemia, and Spec cPL concentrations showed no correlation with creatinine attributing isolated increases to confounding factors such as dehydration [8]. Additionally, in acute kidney injury models, Spec cPL, TLI, and serum lipase activity remained unaffected [33,34].

Pancreatic lipase activity is influenced by production, renal metabolism, and exocrine excretion. Its low molecular weight makes it highly filterable by glomerulus [34]. Consequently, a reduced glomerular filtration rate may contribute to decreased renal clearance and increased serum activity [35]. However, renal handling of lipase is complex and influenced by extra-renal metabolism and tubular function [24]. In the present study, associations were identified between DGGR results and serum creatinine and urea nitrogen concentrations, as well as between DGGR results and total serum lipase activity. These findings suggest that impaired renal function may partially influence lipase measurements in dogs with CKD.

A key limitation in AP diagnosis is the absence of a non-invasive reference test [13,14,27]. False positives indicate that history, clinical examination, and ultrasound alone cannot exclude AP [25], although a recent study reported that mesenteric echogenicity may by a valuable marker for diagnosing AP in dogs [36]. A reference test could refine gray zones and abnormal values in DGGR and SNAP cPL, especially in CKD cases. Pancreatic biopsy, though definitive, is rarely performed due to invasiveness and sampling challenges [8,13,27]. Necropsy studies show high AP prevalence (64%) in dogs dying from various causes, suggesting undiagnosed subclinical AP [37]. Thus, elevated lipase in CKD dogs may reflect impaired clearance, uremic toxin effects, or subclinical AP. Future studies should be performed to compare DGGR and SNAP cPL with a definitive diagnostic method [12,33].

Some limitations apply to our study. First, quantitative Spec cPL, which is considered the most sensitive and specific test for AP in dogs, was not used. Without this reference, we could not directly compare DGGR and SNAP cPL to Spec cPL, limiting our ability to assess their true diagnostic performance in CKD dogs. However, a recent study showed a high correlation between DGGR and spec cPL assays, suggesting that DGGR can reliably quantify pancreatic lipase activity [10]. Additionally, while DGGR and SNAP cPL results were analyzed, the lack of a gold-standard diagnostic test, such as histopathology or post-mortem examination, prevents definitive conclusions regarding the AP status of the dogs included in the present study. Moreover, the possibility of subclinical acute pancreatitis cases among our study dogs cannot be excluded. The measurement of C-reactive protein (CRP) could have helped identify such cases; however, it was not performed because its prognostic value has only recently been reported, and no serum samples are available from our study population [38]. Future studies incorporating quantitative Spec cPL and alternative diagnostic methods will be crucial in refining the diagnostic utility of lipase assays in dogs with CKD. Another limitation of the present study is the small sample size within CKD subgroups, which limits statistical power and reduces the robustness of comparisons among disease stages. Consequently, stage-specific results should be regarded as exploratory.

## 5. Conclusions

This study demonstrates that pancreatic lipase activity can be elevated in dogs with CKD independently of AP. DGGR assay results appear to associate with renal markers and serum lipase activity, whereas SNAP cPL may overestimate pancreatic enzyme elevation in CKD-affected dogs. Applying the DGGR upper reference limit of 216 U/L in clinical practice would reduce the number of samples requiring quantitative Spec cPL measurement, lowering hospitalization costs and allowing for the faster identification of AP, thereby enabling earlier initiation of therapy. This advantage is not available with SNAP cPL, as it is a purely qualitative assay. Therefore, in dogs with CKD suspected of concurrent AP, initial screening with DGGR is recommended. Negative DGGR results can be confidently accepted, while positive results should be interpreted in conjunction with clinical and imaging findings. An accurate assessment of pancreatic lipase in CKD requires integration of clinical, biochemical, and imaging data to avoid the misdiagnosis of AP. DGGR represents a promising quantitative tool for veterinary practice, but further studies are warranted to establish CKD-specific reference ranges and diagnostic thresholds.

## Figures and Tables

**Table 1:** Physical examination findings and symptoms for 25 dogs with chronic kidney disease included in the study recorded at the time of sampling.

Parameter	Clinical Examination Finding/Symptom	*n* ^1^ (%)
Body condition score	1 (poor)	1/25 (4%)
	2 (underweight)	8/25 (32%)
	3 (ideal)	8/25 (32%)
	4 (overweight)	5/25 (20%)
	5 (obese)	3/25 (12%)
Mucous membrane colour	Normal	17/25 (68%)
	Pale	5/25 (20%)
	Anemic	3/25 (12%)
Gastrointestinal symptoms	Diarrhea	4/25 (16%)
	Vomiting	5/25 (20%)
Other clinical symptoms	Depression	2/25 (8%)
	Polyuria	10/25 (40%)
	Polydipsia	12/25 (48%)
	Anorexia	8/25 (32%)
	Fever	0/25 (0%)
Dehydration	None	25/25 (68%)
Abdominal palpation findings	None	25/25 (100%)

^1^ *n* = number of dogs.

**Table 2:** Complete blood count findings for 25 dogs with chronic kidney disease included in the study.

Parameter	Min	Max	Median	IQ = Q75-Q25	Reference Range
HCT (%)	16.2	42	29.9	10.8	37.1–55.0
HGB (g/dL)	4.01	14.1	9.4	4.2	12.0–18.0
WBC (×10^3^/μL)	5.2	27.7	11.5	8.2	6.0–17.0
PLTs (×10^3^/μL)	124	787	312	318.5	200–500

HCT: hematocrit, HGB: hemoglobin concentration, WBC: total white blood cell count, PLT: total platelet count, IQ: interquartile.

**Table 3:** Serum biochemistry and DGGR results for 25 dogs with chronic kidney disease included in the study.

Parameter	Min	Max	Median	IQ = Q75-Q25	Reference Range
TS (g/dL)	5	12.5	8	2.3	5.5–8.0
Albumin (g/dL)	1.2	3.7	2.8	1.1	2.9–4.0
BUN (mg/dL)	54	300	116	76.5	10–38
Creatinine (mg/dL)	1.2	9.6	3.2	2.4	0.7–1.3
Glucose (mg/dL)	75	136	94.5	7.75	65–118
Cholesterol (mg/dL)	126	519	280	128.5	125–296
Triglycerides (mg/dL)	31	149	56	20.5	24–102
ALP (U/L)	32	1081	172	377	32–149
ALT (U/L)	16	220	37	50	18–62
Lipase (U/L)	34	406	86	92.5	5.0–32.0
DGGR (U/L)	5	300	124	220.8	0–125
Phosphate (mg/dL)	3.4	21.5	6.6	5.1	2.2–6
Calcium (mg/dL)	5.5	14.8	9.2	2.3	8.6–10.9
Potassium (mEq/dL)	3.3	6.6	5	0.9	3.7–5.9
Sodium (mEq/dL)	137	169	146	6	144–158

TS: total solids, BUN: blood urea nitrogen, ALP: alkaline phosphatase, ALT: alanine aminotransferase, DGGR: 1,2-o-Dilauryl-rac-glycero-3-glutaric Acid-(6′-methylresorufin) Ester (DGGR)-Lipase IQ: interquartile.

**Table 4:** Variations in kidney function related biochemistry parameters, DGGR results, and main urinalysis findings from 25 dogs with chronic kidney disease included in the study at each of the 4 IRIS stages.

CKD Stage		DGGR (U/L)	CREA (mg/dL)	BUN (mg/dL)	Lipase (mg/dL)	Chol (mg/dL)	ALB (g/dL)	Urine SG	UPC
1	Min	46.5	1.2	66	49	179	2.0	1010	2.4
	Median	80.6	1.4	78	58.5	187.5	2.7	1015	4.0
	Max	114.7	1.6	90	68	196	3.4	1020	5.6
	IQ = Q75-Q25	-	-	-	-	-	-	-	-
	*n* = 2								
2	Min	23.0	1.7	54	37	126	1.2	1010	0.53
	Median	120.2	2.3	111.5	66	245	2.5	1014.5	4.86
	Max	300.0	3.3	175	281	519	3.0	1021	10.5
	IQ = Q75-Q25	136.1	1.1	45.8	87.3	177	1.3	5.7	6.9
	*n* = 6								
3	Min	16.5	2.3	66	34	182	1.9	1010	1.93
	Median	117.1	2.75	124.5	90.5	292.5	2.6	1015.5	6.91
	Max	300.0	3.4	200	160	370	3.7	1020	14.1
	IQ = Q75-Q25	227.9	0.9	52.3	79.5	153.3	1.1	9	7.8
	*n* = 8								
4	Min	5.0	4.3	87	39	225	1.5	1010	0.7
	Median	209.9	6.8	162	118	319	3.1	1012	4.75
	Max	300.0	9.6	300	406	428	3.6	1020	14
	IQ = Q75-Q25	235.5	3.7	147.5	248.5	137.5	0.7	3	8.4
	*n* = 9								

CKD: Cchronic kidney disease, DGGR: 1,2-o-Dilauryl-rac-glycero-3-glutaric Acid-(6′-methylresorufin) Ester (DGGR)-Lipase, CREA: creatinine, BUN: blood urea nitrogen, CHOL: cholesterole, ALB: albumin, SG: specific gravity, UPC: urine protein-to-creatinine ratio, *n*: number of dogs. IQ: interquartile.

**Table 5:** Serum pancreatic lipase levels of 25 dogs with chronic kidney disease categorized as normal and abnormal based on a quantitative (DGGR) and a qualitative (SNAP cPL) assay.

	SNAP cPL Normal Result	SNAP cPL Abnormal Result	*n*
**DGGR measured normal pancreatic lipase activity (<125 IU/dL)**	7	6	13
**DGGR measured abnormal pancreatic lipase activity (≥125 IU/dL)**	0	12	12
*n*	7	18	

DGGR: 1,2-o-Dilauryl-rac-glycero-3-glutaric Acid-(6′-methylresorufin) Ester (DGGR)-Lipase, cPL: canine pancreatic lipase, *n*: number of dogs.

**Table 6:** Serum pancreatic lipase results for 25 dogs with chronic kidney disease (CKD) across 4 CKD stages categorized as normal and abnormal based on a quantitative (DGGR) and a qualitative (SNAP cPL) assay.

CKD Stage	1	2	3	4	*n*
DGGR measured normal pancreatic lipase activity (<125 IU/dL)	2	3	4	4	13
DGGR measured abnormal pancreatic lipase activity (≥125 IU/dL)	0	3	4	5	12
SNAP cPL normal result	2	1	3	1	7
SNAP cPL abnormal result	0	5	5	8	18
*n*	2	6	8	9	

CKD: chronic kidney disease, DGGR: 1,2-o-Dilauryl-rac-glycero-3-glutaric Acid-(6′-methylresorufin) Ester (DGGR)-Lipase, cPL: canine pancreatic lipase, *n*: number of dogs.

## Data Availability

The original contributions presented in this study are included in the article. Further inquiries can be directed to the corresponding author.

## Abbreviations

The following abbreviations are used in this manuscript:

CKD	Chronic kidney disease
DGGR	1,2-o-dilauryl-rac-glycero-3-glutaric acid-(6′-methylresorufin) ester
AP	Acute pancreatitis
CBC	Complete blood count
IRIS	International Renal Interest Society
